# Estimation of Static Lung Volumes and Capacities From Spirometry Using Machine Learning: Algorithm Development and Validation

**DOI:** 10.2196/65456

**Published:** 2025-03-24

**Authors:** Scott A Helgeson, Zachary S Quicksall, Patrick W Johnson, Kaiser G Lim, Rickey E Carter, Augustine S Lee

**Affiliations:** 1 Division of Pulmonary and Critical Care Medicine, Mayo Clinic, 4500 San Pablo Road S, Jacksonville, FL, 32224, United States, 1 9049532000; 2 Digital Innovation Laboratory, Department of Quantitative Health Sciences, Mayo Clinic, Jacksonville, FL, United States; 3 Division of Pulmonary and Critical Care Medicine, Mayo Clinic, Rochester, MN, United States

**Keywords:** artificial intelligence, machine learning, pulmonary function test, spirometry, total lung capacity, AI, ML, lung, lung volume, lung capacity, spirometer, lung disease, database, respiratory, pulmonary

## Abstract

**Background:**

Spirometry can be performed in an office setting or remotely using portable spirometers. Although basic spirometry is used for diagnosis of obstructive lung disease, clinically relevant information such as restriction, hyperinflation, and air trapping require additional testing, such as body plethysmography, which is not as readily available. We hypothesize that spirometry data contains information that can allow estimation of static lung volumes in certain circumstances by leveraging machine learning techniques.

**Objective:**

The aim of the study was to develop artificial intelligence-based algorithms for estimating lung volumes and capacities using spirometry measures.

**Methods:**

This study obtained spirometry and lung volume measurements from the Mayo Clinic pulmonary function test database for patient visits between February 19, 2001, and December 16, 2022. Preprocessing was performed, and various machine learning algorithms were applied, including a generalized linear model with regularization, random forests, extremely randomized trees, gradient-boosted trees, and XGBoost for both classification and regression cohorts.

**Results:**

A total of 121,498 pulmonary function tests were used in this study, with 85,017 allotted for exploratory data analysis and model development (ie, training dataset) and 36,481 tests reserved for model evaluation (ie, testing dataset). The median age of the cohort was 64.7 years (IQR 18‐119.6), with a balanced distribution between genders, consisting 48.2% (n=58,607) female and 51.8% (n=62,889) male patients. The classification models showed a robust performance overall, with relatively low root mean square error and mean absolute error values observed across all predicted lung volumes. Across all lung volume categories, the models demonstrated strong discriminatory capacity, as indicated by the high area under the receiver operating characteristic curve values ranging from 0.85 to 0.99 in the training set and 0.81 to 0.98 in the testing set.

**Conclusions:**

Overall, the models demonstrate robust performance across lung volume measurements, underscoring their potential utility in clinical practice for accurate diagnosis and prognosis of respiratory conditions, particularly in settings where access to body plethysmography or other lung volume measurement modalities is limited.

## Introduction

Pulmonary function testing (PFT) provides physiological measurements of the respiratory system across multiple dimensions, typically classified into (1) spirometry, which measures air flow, lung volumes, and capacities during a expiratory forced vital capacity (FVC) maneuver; (2) static lung volumes; and (3) gas exchange parameters such as the diffusing capacity for carbon monoxide and oxygen saturations [1]. PFTs are critical for the diagnosis and prognostication of respiratory disorders, and provide a noninvasive method for measuring and monitoring the degree of respiratory impairment [2]. They are recommended for the initial evaluation of patients with chronic dyspnea and other respiratory symptoms, as well as for individuals at risk of respiratory complications due to transplant or surgery [3,4].

Basic spirometry remains the most widely used component of PFT, largely due to its size and portability, allowing it to be performed in clinic office settings or remotely at home with adequate training. However, spirometry, by definition is an expiratory FVC maneuver that focuses on assessing airflow limitations and does not directly measure static lung volumes, which can be integral to understanding many respiratory conditions [4]. Accurate determination of static lung volumes traditionally necessitates more complex and resource-intensive techniques such as body plethysmography or gas dilution methods, with body plethysmography serving as the current gold standard [3,5,6]. However, these methods, while precise, may not always be readily accessible, cost-effective, or suitable for routine clinical practice outside a specialized pulmonary function laboratory.

Advancements in artificial intelligence (AI) techniques have introduced new avenues in health care, offering the potential to derive comprehensive insights from existing data, including patterns not easily recognizable through human interpretation or standard statistical modeling. A prior study by Beverin et al [7] examined the prediction of total lung capacity from spirometry using three tree-based machine learning (ML) models, achieving a mean squared error of 560.1 mL. They further developed models to classify restrictive ventilatory impairment, achieving a sensitivity and specificity of 83% and 92%, respectively. However, they did not explore prediction of the complete lung volume assessments. Predicting functional residual capacity status, for example, could facilitate the prevention of atelectasis during anesthesia [8]. Another study by Evankovich et al [9] developed a regression model in patients with chronic obstructive pulmonary disease (COPD) to predict residual volume and its elevation status, achieving an area under the receiver operating characteristic curve (ROC) of 0.95 for predicting residual volume above 175%. However, these models lack applicability beyond the COPD cohort [9]. Given this context, we hypothesized that ML models could predict static lung volumes using spirometry alone across a diverse cohort of lung conditions. Such an approach could reduce the need for identifying those who would benefit most from formal lung volume assessments. In this study, we applied ML approaches to develop and validate an algorithm for estimating lung volumes and capacities from standard spirometry. We further examined the model performance among subsets of physiologic derangements such as obstructive and restrictive ventilatory disorders.

## Methods

### Cohort Selection

This study was approved by the Institutional Review Board (20‐009821) with a waiver of consent. The dataset curated for this study was obtained from the Mayo Clinic PFT database, which houses PFT data from two distinct US regions (Midwest and Southeast), with records from February 19, 2001, to December 16, 2022. The PFTs performed on the same day—with paired spirometry and lung volume data, without the use of methacholine or a bronchodilator—were identified. Individuals under 18 years of age and patients who opted out of authorizing their data for research use were excluded from the analysis. All lung volume measurements were performed using body plethysmography. For models trained to classify normal versus abnormal lung volume measures, an additional requirement was applied to ensure nonmissing demographics within the boundaries of the Global Lung Initiative GLI2021 lung volume estimation equations [10]. If an individual underwent multiple PFTs, only their most recent PFT measurement comprising both lung volumes and spirometry was used. The following lung volume measures were selected for prediction: expiratory reserve volume (ERV), functional residual capacity (FRC), residual volume (RV), total lung capacity (TLC), the ratio of RV to TLC as a percentage (RV/TLC), and vital capacity (VC).

### Preprocessing

Following the initial database query, the dataset was augmented with reference lung function measures for both spirometry and lung volume measures, including the lower limit of normal function (LLN), the upper limit of normal function (ULN), and the expected volume. These values were generated using a custom package built according to the Global Lung Initiative pulmonary function testing reference equation publications [1,11,12]. The LLN and ULN values were used to assign “normal” (within the LLN/ULN range) or “abnormal” (below LLN or above ULN) status to reformulate the lung volume regression problem into a classification task.

Both the regression and classification data sets were split into independent training and testing subsets using a randomized 70/30 split before any downstream exploratory analysis or model development. Features provided to the models included forced expiratory volume in the first second of exhalation (FEV1), forced vital capacity (FVC), the ratio of FEV1 and FVC (FEV1/FVC), peak expiratory flow, estimated maximum vital capacity, age, gender, height, weight, and race (White, African American, Northeast Asian, Southeast Asian, and Other).

### Model Selection and Evaluation

A randomized grid search was performed using various ML algorithms, including a generalized linear model with regularization, distributed random forests, extremely randomized trees, gradient-boosted trees, and XGBoost. Models were tuned using appropriate parameter grids via five-fold cross-validation on the training dataset to provide estimates of performance summarized using applicable metrics, including root mean squared error (RMSE) for regression and area under the receiver operating characteristic curve (ROC-AUC) for classification [13]. Final tuning parameters were selected from the candidate model with the highest cross-validation performance (lowest RMSE for regression, highest ROC-AUC for classification), which was ranked highest among all explored configurations. The model was then refitted to the full training data set using the chosen hyperparameters before evaluation on the testing dataset (Multimedia Appendix 1). For the classification models, the probability threshold was selected to maximize the Youden index on the training data set.

The regression model performance was evaluated visually using prediction scatter plots and summary metrics, including RMSE, mean absolute error (MAE), mean signed difference, mean percentage error (MPE), mean absolute percentage error (MAPE), and the correlation-based coefficient of determination [14]. The classification model was evaluated with the area under the receiver-operating-characteristic curve (AUC), accuracy , sensitivity (SENS), specificity, positive predictive value, negative predictive value (NPV), precision, recall, positive likelihood ratio (LRT+), negative likelihood ratio (LRT-), odds ratio, and F1-score. All modeling was performed using the H2O AutoML cluster (version 3.44.0.3) [15]. Further details regarding the grid search process, parameter tuning, and model implementation are available in the H2O official documentation [15] (Multimedia Appendix 2).

In the cohort summary tables, categorical data were displayed as counts and percentages, while continuous data were displayed as medians and ranges. Standardized mean differences were computed to identify significant differences in variables between the training and testing datasets, with insignificant differences defined as a value <0.1. The regression and classification models were applied to the specific PFT patterns (normal, obstructed, restricted, and mixed pattern) defined by the American Thoracic Society (ATS) [10]. All analyses were performed using R software (version 4.2.2; R Foundation for Statistical Computing) on a Google Cloud Platform virtual machine.

### Ethical Considerations

This study was approved by the Mayo Clinic Institutional Review board (22-009471) and was determined to be exempt (45 CFR 46.104d, Category 4). All data was deidentified for this study, and no compensation was provided to the participants

## Results

A total of 121,498 PFTs were used in this study, with 85,017 allocated for exploratory data analysis and model development and 36,481 tests reserved for model evaluation. The median age across the cohort was 64.7 years (IQR 18‐119.6), with a nearly balanced gender distribution between genders, with 48.2% (n=58,607) female patients and 51.8% (n=62,889) male patients. The cohort was predominantly White (n= 114,388, 94.1%), followed by African American patients (n=4,656, 3.8%). Of particular importance, the distribution of baseline PFT measures—both spirometry and lung volumes—showed no differences between the training and testing datasets. Standardized mean differences, indicating the degree of difference between the training and testing sets, were minimal across all variables, suggesting a well-balanced model development and testing cohorts. A complete breakdown is provided in Table 1.

**Table 1.** Cohort summary.

Variables	Training dataset (n=85,015)	Testing dataset (n=36,481)	Total (N=121,496)	Standardized difference
Age (years), median (IQR)	64.7 (18.0-119.6)	64.7 (18.0-101.0)	64.7 (18.0-119.6)	.005
Gender, n (%)				.004
Female	40,964 (48.2)	17,643 (48.4)	58,607 (48.2)	
Male	44,051 (51.8)	18,838 (51.6)	62,889 (51.8)	
Race, n (%)				.01
White	80,048 (94.2)	34,340 (94.1)	114,388 (94.1)	
African American	3223 (3.8)	1433 (3.9)	4656 (3.8)	
Southeast Asian	508 (0.6)	213 (0.6)	721 (0.6)	
Northeast Asian	64 (0.1)	27 (0.1)	91 (0.1)	
Other	1172 (1.4)	468 (1.3)	1640 (1.3)	
Height (m), median (IQR)	1.7 (0.5-2.2)	1.7 (0.2-2.0)	1.7 (0.2-2.2)	.001
Weight (kg), median (IQR)	82.8 (7.8-253.4)	82.9 (12.9-400.0)	82.8 (7.8, 400.0)	.001
ATS^a^ Pattern, n (%)				.007
Normal	33,150 (41.2)	14,346 (41.6)	47,496 (41.3)	
Obstruction	16,810 (20.9)	7173 (20.8)	23,983 (20.9)	
Restriction	19,856 (24.7)	8482 (24.6)	28,338 (24.7)	
Mixed defect	10,611 (13.2)	4512 (13.1)	15,123 (13.2)	
PFT^b^ measures, median (IQR)				
FEV1^c^	2.0 (0.2-6.8)	2.0 (0.2-6.1)	2.0 (0.2-6.8)	.005
FVC^d^	2.9 (0.3-8.8)	2.9 (0.5-8.3)	2.9 (0.3-8.8)	.004
FEV1/FVC^e^	71.6 (16.2-100.0)	71.5 (16.2-100.0)	71.6 (16.2-100.0)	.002
PEF^f^	6.1 (0.7-18.8)	6.2 (0.6-17.5)	6.2 (0.6-18.8)	.001
VC (Spiro)^g^	2.9 (0.3-8.8)	2.9 (0.5-8.3)	2.9 (0.3-8.8)	.004
RV^h^	2.3 (0.0-11.8)	2.3 (0.1-10.4)	2.3 (0.0-11.8)	.003
TLC^i^	5.5 (0.9-13.9)	5.5 (1.3-13.1)	5.5 (0.9-13.9)	.004
RV/TLC^j^	43.6 (1.2-90.7)	43.6 (3.4-89.7)	43.6 (1.2-90.7)	.002
FRC^k^	3.2 (0.5-12.3)	3.2 (0.4-10.8)	3.2 (0.4-12.3)	.004
ERV^l^	0.8 (0.0-4.4)	0.8 (0.0-4.1)	0.8 (0.0-4.4)	.003
VC (Pleth)^m^	3.0 (0.3-8.8)	3.0 (0.5-8.4)	3.0 (0.3-8.8)	.003

a ATS: American Thoracic Society.

b Pulmonary function test.

c FEV1: Forced expiratory volume in the first second.

d FVC: Forced vital capacity.

e FEV/FVC: Ratio of FEV1 to FVC (as a percentage).

f PEF: Peak expiratory flow.

g VC (Spiro): Vital capacity measured via spirometry.

h RV: Residual volume.

i TLC: Total lung capacity.

j RV/TLC: Ratio of RV to TLC (as a percentage).

k FRC: Functional residual capacity.

l ERV: Expiratory reserve volume.

m VC (Pleth): Vital capacity measured via body plethysmography.

Multimedia Appendix 3 stratifies the same cohort according to the ATS classification criteria for pulmonary function patterns (ie, normal, obstructive, restrictive, and mixed pattern). This stratification highlights differences in demographics and pulmonary function measures between individuals with normal, obstructive, restrictive, or mixed patterns assigned using spirometry. Predictably, spirometry measures—including FEV1, FVC, and the FEV1/FVC ratio—significantly differed between groups (*P* values<.001), as did all phenotype-related parameters presented in the table.

### Lung Volume Regression

The final models chosen for evaluation were selected based on the lowest RMSE values and varied minimally in type across the lung volumes of interest. XGBoost models were identified as the best approach for predicting all lung volumes except TLC, for which traditional gradient-boosted trees showed superior performance.

**Figure 1.**
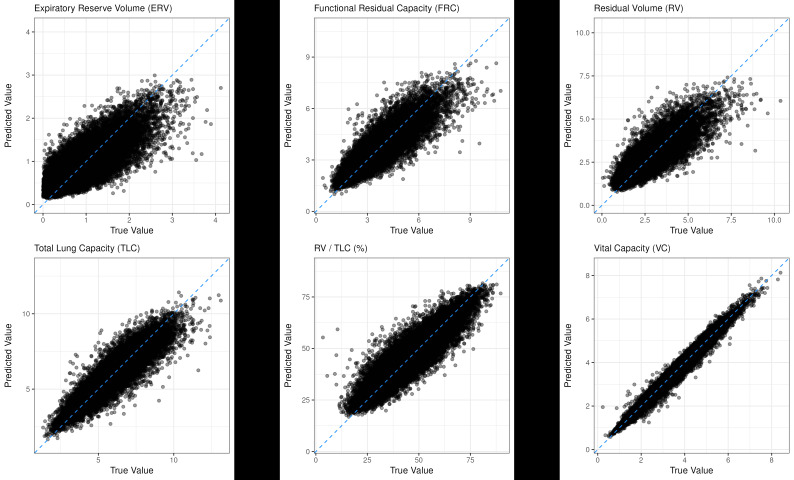
Regression scatter plots of predicted versus true lung volume measures.

Model metrics were similar between the training and testing cohorts, suggesting a reasonable trade-off between overfitting and underfitting during model training (Table 2). Findings showed a strong performance overall, with relatively low RMSE and MAE values observed across all predicted lung volumes. MPE showed a negative skew across all lung volumes. However, quantile-quantile plot analyses showed that predicted values closely followed a theoretical normal distribution, with slight underprediction and overprediction of high and low values at the extremes, respectively. Paired with mean signed differences of zero—also known as the mean bias error—these evaluations suggest no global bias in the direction of model predictions. Instead, these skewed MPE values were the result of extreme values at the tails of the distribution. A complete breakdown of model performance metrics is presented in Table 2, with complementary prediction scatter plots in Figure 1. Further subgroup analysis with different ATS patterns showed relatively similar results overall and across all categories in Multimedia Appendix 2).

**Table 2.** Regression model performance metrics.

Variables	Training dataset						Testing dataset					
Volume	RMSE (L)^a^	MAE^b^	MSD (L)^c^	MPE (%)^d^	MAPE (%)^e^	RSQ^f^	RMSE (L)	MAE	MSD (L)	MPE (%)	MAPE (%)	RSQ
Expiratory Reserve Volume (ERV)	0.31	0.24	0	−40.12	60.28	0.64	0.33	0.25	0.00	−39.10	59.95	0.61
Functional Residual Capacity (FRC)	0.56	0.42	0	−2.83	12.93	0.78	0.59	0.44	0.00	−2.91	13.51	0.75
Residual Volume (RV)	0.54	0.40	0	−4.86	17.29	0.73	0.56	0.41	0.00	−4.92	17.80	0.71
RV / TLC	5.07	3.93	0	−1.61	9.55	0.82	5.20	4.03	0.03	−1.58	9.83	0.81
Total Lung Capacity (TLC)	0.55	0.41	0	−1.07	7.57	0.87	0.58	0.43	0.00	−1.10	7.92	0.85
Vital Capacity (VC)	0.15	0.11	0	−0.27	3.73	0.98	0.15	0.11	0.00	−0.33	3.91	0.98

a Root mean squared error.

b Mean absolute error.

c Mean signed deviation.

d Mean percent error.

e Mean absolute percent error.

f R-Squared.

### Lung Volume Classification

Due to limitations in demographic information (ie, age and race) required for the calculation of LLN and ULN boundaries, a total of 114,377 PFTs from the regression cohort were successfully recharacterized for the development of classification models, with 34,314 PFTs reserved for model evaluation. A comparison of demographics, spirometry, and lung volumes between the training and testing data sets can be seen in MultimediaAppendices 5  6. These tables mirror the factors presented in Table 1, except for the lung volume classes (normal vs abnormal), which are unique to this subset.

**Figure 2.**
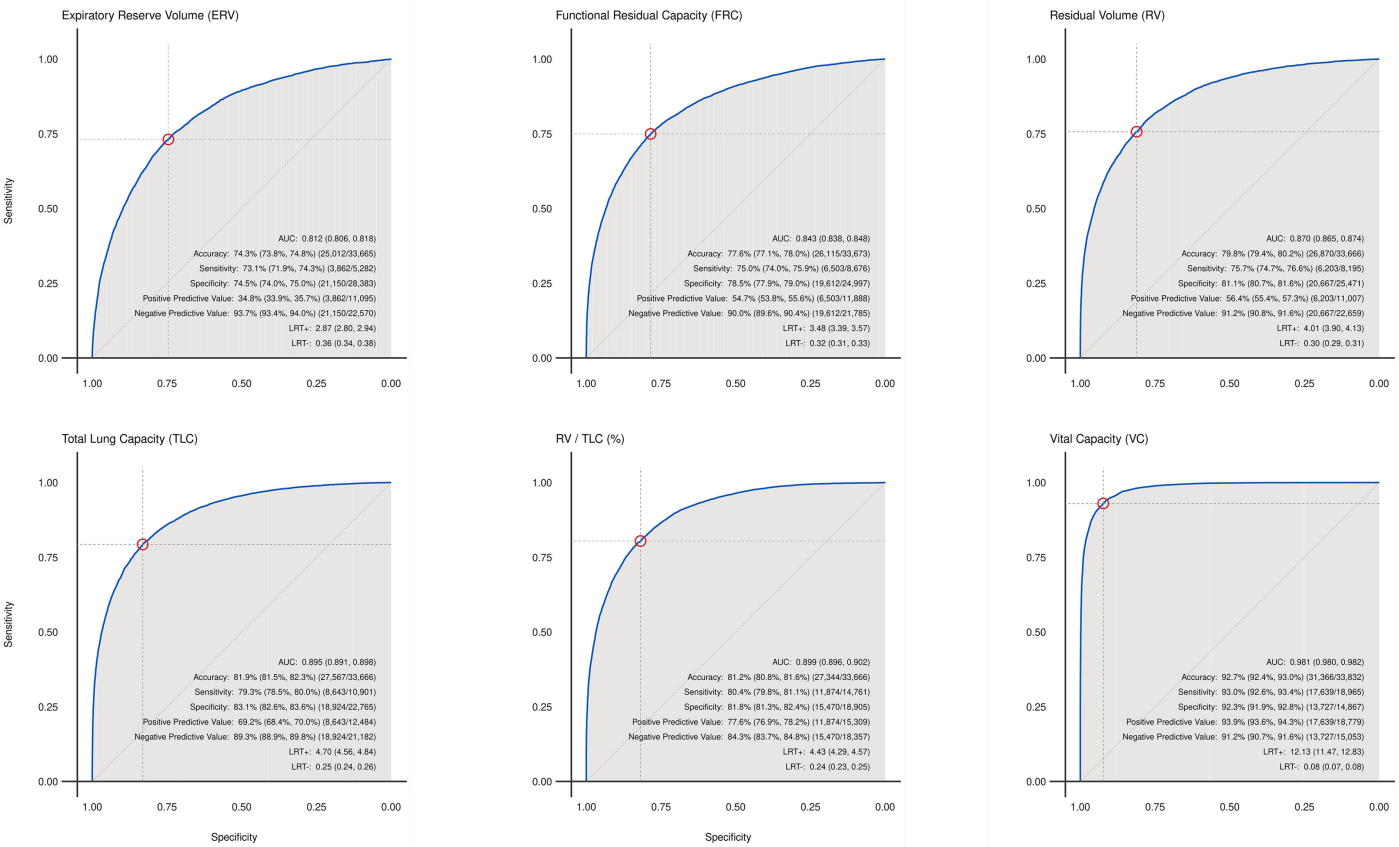
Classification receiver operating characteristic (ROC) curves.

Similar to the regression tasks, the final classification models selected for downstream evaluation varied minimally in type across lung volumes and were selected based on the largest ROC-AUC values. Traditional gradient-boosted trees ranked best for classifying lung volume status for FRC and vital capacity. XGBoost models ranked at the top for all other lung volume classifications. Across all lung volume categories, the models demonstrated strong discriminatory capacity, as indicated by high AUC values ranging from 0.85 to 0.99 in the training dataset and 0.81 to 0.98 in the testing dataset. High accuracy scores, ranging from 0.74 to 0.93, illustrate the ability of each model to correctly classify instances overall, with sensitivity scores ranging from 0.73 to 0.93 in the testing data set, indicating the effectiveness in identifying positive cases (ie, lung volume measurements outside the expected normal range). The high NPVs (ranging from 0.84 to 0.94) highlight each model’s ability to correctly identify normal lung volumes. The greater variation in positive predictive value across the lung volume classes (ranging from 0.35‐0.94) suggests that some models may struggle to identify positive cases correctly, relative to the larger population of normal test findings. Classification performance metrics can be found in Table 3, with complementary ROC curves in Figure 2.

**Table 3.** Classification model performance metrics.

Volume	Training dataset										Testing dataset									
	AUC^a^	ACC^b^	SENS^c^	SPEC^d^	PPV^e^	NPV^f^	LRT+^g^	LRT-^h^	OR^i^	F1^j^	AUC	ACC	SENS	SPEC	PPV	NPV	LRT+	LRT-	OR	F1
Expiratory reserve volume (ERV)	0.85	0.76	0.78	0.76	0.38	0.95	3.24	0.29	11.23	0.51	0.81	0.74	0.73	0.75	0.35	0.94	2.87	0.36	7.95	0.47
Functional residual capacity (FRC)	0.88	0.80	0.79	0.80	0.58	0.92	3.99	0.26	15.16	0.67	0.84	0.78	0.75	0.78	0.55	0.90	3.48	0.32	10.90	0.63
Residual volume (RV)	0.90	0.82	0.80	0.83	0.60	0.93	4.70	0.24	19.89	0.69	0.87	0.80	0.76	0.81	0.56	0.91	4.01	0.30	13.40	0.65
RV/TLC (%)	0.91	0.82	0.82	0.83	0.78	0.86	4.77	0.22	21.60	0.80	0.90	0.81	0.80	0.82	0.78	0.84	4.43	0.24	18.52	0.79
Total lung capacity (TLC)	0.93	0.85	0.84	0.85	0.73	0.92	5.71	0.19	30.77	0.78	0.89	0.82	0.79	0.83	0.69	0.89	4.70	0.25	18.86	0.74
Vital capacity (VC)	0.99	0.95	0.95	0.94	0.95	0.94	16.59	0.05	309.54	0.95	0.98	0.93	0.93	0.92	0.94	0.91	12.13	0.08	160.18	0.93

a AUC: area under the receiver operating curve.

b ACC: accuracy.

c SENS: sensitivity.

d SPEC: specificity.

e PPV: positive predictive value.

f NPV: negative predictive value.

g LRT+: likelihood ratio test+.

h LRT–: likelihood ratio test-.

i OR: odds ratio.

j F1: F1-score.

When stratified by PFT patterns, unique strengths, and weaknesses were observed across subgroups (Multimedia Appendix 7). These variations can be attributed to the limitations of the training data, feature space, and models, while others were driven by the rarity of certain lung volume abnormalities in specific spirometry-defined patterns. For instance, in classifying ERV status—arguably the most challenging lung volume explored in this study—the model showed consistently high NPVs across all spirometry pattern types, highlighting general confidence in predicting normal lung volume status. However, it achieved notably better sensitivity in the “restriction” and “mixed pattern” subsets (0.91 and 0.75). Comparing these sensitivities and other metrics to those in the “normal” and “obstruction” subgroups, the model seems to struggle to detect positive cases in patients with normal or obstructive spirometry findings.

## Discussion

The development of ML models to predict lung volume status (normal vs abnormal findings) from spirometry in over 110,000 patients has yielded highly encouraging results, displaying remarkable discriminatory power with high AUC values (0.81‐0.95) across measured lung volumes. Estimates of FRC, TLC, RV, and the RV/TLC ratio status show strong sensitivity and specificity. These metrics remain largely consistent across spirometry-defined pattern subgroups, with a few exceptions that can generally be attributed to the rarity of abnormal lung volume measures in certain spirometry patterns. The ability to predict lung volume measures without having to perform extensive testing represents a promising innovation for improving the diagnosis and management of dyspnea and chronic respiratory diseases, particularly in the primary care setting [16]. The strong predictive performance of lung volume measurement underscores the potential of these models as a transformative tool in respiratory medicine, offering substantial clinical implications and opportunities for enhancing patient care.

The performance of the regression models showed a high correlation between the training and testing datasets, suggesting that the models were able to effectively capture the relationship between spirometry-derived features and measured lung volumes and capacities derived from body plethysmography. The effectiveness of the models was evident in their ability to closely approximate lung volumes with minimal deviation from true values on average. The RMSE and MAE values are low relative to their respective lung volume ranges. For instance, the median TLC measure in the cohort was 5.5 L, with the model attaining an MAE of 0.43 L and an MAPE of 7.92%. The ability to accurately estimate the RV/TLC ratio further highlights the potential of these models in capturing the dynamic interplay between these volumes, which is particularly relevant in differentiating between common lung conditions such as COPD, asthma, and restrictive lung diseases [17-20]. The high R-squared values observed for TLC (0.87 in the training set and 0.85 in the testing set) underscore the model’s capacity to capture a significant portion of the variance in TLC measurement. Similarly, the robust estimation of RV (R-squared of 0.73 in the training set and 0.71 in the testing set) and FRC (R-squared of 0.78 in the training set and 0.75 in the testing set) further validates model reliability in estimating lung volumes crucial for the evaluation of respiratory function. The model demonstrated a high correlation for vital capacity (*R*^2^=0.98). However, this finding is misleading, as spirometry already provides an accurate estimate of vital capacity, making it trivial to map to a similar value obtained via body plethysmography, assuming minimal measurement error and consistent effort on the part of the patient when executing breathing maneuvers. A significant change in TLC has been reported to be 10% over one year, whereas this model was able to predict TLC within 7.5% and 550 mL [10]. No significant changes were reported in FRC or RV over time. Considering the performance metrics as a whole, the potential of these models to augment clinical practice is encouraging, with R-squared values exceeding 0.7 for all volumes except ERV, which seems to be the most challenging volume to predict accurately. Estimation of TLC, RV, and their ratio (RV/TLC) is particularly promising, as the accurate estimation of the RV/TLC ratio facilitates the identification of air trapping and hyperinflation, which are key factors in many patients’ symptomatology [3,17-20]. Moreover, the reasonable estimation of FRC suggests its potential utility as an indicator for restrictive lung disease diagnosis and treatment. This is particularly important as body plethysmography directly measures only FRC, which is then used to calculate the other variables.

Focusing on the estimation of ERV, the notably high MAPE indicates a relatively subpar overall performance. Given that ERV has the narrowest range of measured values (ie, median 0.8 L, (IQR 0-44) L and a large RMSE of 0.31 relative to the ERV range, this elevated MAPE may be partially influenced by the smaller margin for error [21]. ERV measures the volume of air that an individual can exhale after completing a normal tidal breath. Pairing this with spirometry, individuals with a higher ERV may experience more difficulty with exhalation or exhibit an obstructive pattern on spirometry with a lower FEV1 measure [22,23]. A higher ERV could be a sign of lung hyperinflation, while other factors like obesity, pregnancy, and significant ascites can decrease ERV [22,24]. Lung hyperinflation in obstructed patients, which is defined as elevated FRC, RV, RV/TLC, or occasionally ERV, is highly variable in patients and occurs inconsistently over time [23,25]. This inconsistency, combined with ERV’s narrow range, makes it challenging to predict.

Highlighting a more robust model, predictions for the RV/TLC ratio are strong overall, with AUC values ranging from 0.8 to 0.86 across all patterns and 0.91 in the full cohort. Except for normal pattern PFTs, the model consistently achieved sensitivities >0.84, but it struggled to identify positive cases in normal spirometry tests. While spirometry alone does not directly measure RV or TLC, FEV1 and FVC can indirectly reflect changes in lung volumes. In obstructive lung diseases, a reduction in FEV1/FVC ratio combined with an increase in the RV/TLC ratio often indicates air trapping [22-25]. In restrictive diseases, such as pulmonary fibrosis, spirometry may show decreased FVC with a preserved or decreased RV/TLC ratio, suggesting reduced air trapping [22-25]. Given the absence of abnormal FEV1 and FVC values, normal spirometry patterns would not usually suggest the existence of an abnormal RV/TLC ratio, potentially explaining the reduced sensitivity to predicting abnormal RV/TLC in normal spirometry.

A previous study used a CatBoost model to predict the TLC from spirometry, yielding good results [7]. The study reports an MSE of 560.1 mL for TLC and a positive predictive value for reduced TLC of 8% or 67%, depending on the model parameters. However, this study only focused on TLC and did not assess other pulmonary physiologic parameters obtained through lung volume measurements, such as FRC and RV. These parameters are necessary as they are crucial for assessing prognosis in various respiratory diseases [26-30].

Several studies have highlighted the importance of lung volume assessments for the diagnosis and prognosis of respiratory diseases [31]. In routine practice, it can aid in the early detection, diagnosis, and monitoring of respiratory conditions such as COPD, restrictive lung diseases, and neuromuscular disorders affecting respiratory function [10,32,33]. For instance, lung volume measurements (specifically, FRC and TLC) strongly correlate with mortality risk among patients with idiopathic pulmonary fibrosis [27,28,30]. This illustrates that the prediction of lung volumes from traditional spirometry holds substantial promise in clinical scenarios where lung volume measurements cannot be directly performed, such as primary care offices, or health care facilities in rural areas where the equipment for measuring lung volumes is not readily accessible. Another scenario is when a patient is not capable of physically performing lung volume measurements, which could involve physical conditions that prevent them or any number of other limitations that could potentially limit them. Additionally, it may facilitate personalized treatment plans by providing a more nuanced understanding of a patient’s lung capacities, as lung volume measurements are typically performed only after a patient is determined to have an abnormal spirometry, unless in specialized centers.

Accurate assessment of lung volumes is pivotal in diagnosing and monitoring various respiratory conditions, including COPD, interstitial lung diseases, neuromuscular disorders, and restrictive lung diseases [4,32]. If lung volume measurements are not performed, vital capacity is often used as a surrogate [34,35]. However, there is a significant error in the application of this method, as a reduced vital capacity can be seen in restrictive lung disease and obstructive lung disease with increased residual volume [36]. A restrictive defect on lung volume measurements has rarely been seen occurring with normal vital capacity, and approximately 58% of the time with low vital capacity measurements [36]. Another study showed that when forced vital capacity >100% predicted in males or >85% predicted in females ruled out a restrictive pattern on lung volumes [37]. The use of direct lung volume prediction models, such as those developed in this study, have a significantly better performance than those used in these prior studies and could reduce the frequency of clinical scenarios where lung volumes are unknown.

The AI model’s ability to estimate lung volumes from readily available spirometry data streamlines these diagnostic procedures. A typical spirometry test may take approximately 30‐45 minutes, while lung volume measurements add another 15‐30 minutes [38,39]. Replacing or complementing traditional, more resource-intensive lung volume measurement techniques with the AI model’s predictions from spirometry data offers cost-effective alternatives. The physician fee for spirometry ranges from $29.62 to $150.68, depending upon the medications used, while measuring lung volumes adds another $59.98 to the cost [40]. This approach optimizes healthcare resources, reduces patient burden associated with additional tests, and potentially increases the efficiency of healthcare delivery.

The accessibility of spirometry in various healthcare settings, coupled with the estimation of both lung volumes via the developed models, opens avenues for telemedicine applications. Remote monitoring and assessment of spirometry are already being performed and could be facilitated and enhanced with automated decision support systems utilizing models such as those developed in this study [41-43]. Such strategies could enable the continuous monitoring of patients with chronic respiratory conditions that affect lung volumes [41-43]. This aligns with the evolving landscape of telemedicine, emphasizing its potential in respiratory care.

Despite the remarkable performance of the predictive models, certain limitations warrant consideration. Model training and testing relied on datasets with potential biases in demographic variables, including a majority-White population (91%) of older adults (median age 64.7) years. These factors potentially limit the generalizability to diverse populations, although this model was developed with patients of all ages from two distinct regions of the United States (Midwest and Southeast). Further validation across broader demographic groups from various clinical settings is essential to establish widespread applicability and reliability. Moreover, continuous refinement and validation of the models using larger datasets encompassing a broader spectrum of respiratory conditions and disease severities is imperative. This iterative process would enhance model performance while preventing model drift, ensuring its efficacy in diverse clinical scenarios even as standard clinical practices are updated or changed.

In conclusion, the development of AI models for predicting lung volumes from spirometry represents an advancement in pulmonary function assessment. The remarkable sensitivity and specificity offered by the classification models affect a transformative approach to complement traditional lung volume measurement techniques. While the regression models may not attain the same level of performance, the continuous nature of their estimates provides a unique addition to supplement and contextualize binary classifications, potentially elucidating new insights into the remote monitoring of pulmonary function. If integrated into clinical practice, these models hold the promise of revolutionizing respiratory care, enabling more comprehensive and accessible assessments of lung function, and ultimately improving patient outcomes. Overall, the models demonstrate robust performance across lung volume measurements, underscoring their potential utility in clinical practice for accurate diagnosis and prognosis of respiratory conditions in locations where access to body plethysmography or other lung volume measurement modalities is challenging..

## Supplementary material

10.2196/65456 Multimedia Appendix 1 Classification model parameters.

10.2196/65456 Multimedia Appendix 2 Regression model parameters.

10.2196/65456 Multimedia Appendix 3 Regression model cohort summary.

10.2196/65456 Multimedia Appendix 4 Classification model cohort summary.

10.2196/65456 Multimedia Appendix 5 Classification model cohort summary by American Thoracic Society patterns.

10.2196/65456 Multimedia Appendix 6 Regression model performance metrics.

10.2196/65456 Multimedia Appendix 7 Classification model performance metrics.

## Abbreviations:

AI: artificial intelligence
AUC: area under the receiver-operating-characteristic curve
COPD: chronic obstructive pulmonary disease
ERV: expiratory reserve volume
FEV1: forced expiratory volume in the first second of exhalation
FEV1/FVC: ratio of FEV1 and FVC
FRC: functional residual volume
FVC: forced vital capacity
LLN: lower limit of normal
LRT+: positive likelihood ratio
LRT-: negative likelihood ratio
MAE: mean absolute error
MAPE: mean absolute percentage error
ML: machine learning
MPE: mean percentage error
NPV: negative predictive value
PFT: pulmonary function test
PPV: positive predictive value
RMSE: root mean squared error
RV: residual volume
RV/TLC: ratio of residual volume to total lung capacity
SPEC: specificity
TLC: total lung capacity
ULN: upper limit of normal
VC: vital capacity
