# Supplementary material for: Estimation of Static Lung Volumes and Capacities From Spirometry Using Machine Learning: Algorithm Development and Validation
Source: JMIR AI. 2025 Mar 24;4:e65456. doi: 10.2196/65456 (PMC12223454; doi:10.2196/65456)
Supplement: Multimedia Appendix 2 [file ai-v4-e65456-s002.docx]

| **Lung Volume** | **Model Type** | **col_sample_rate^a^** | **col_sample_rate_per_tree^b^** | **max_depth^c^** | **min_rows^d^** | **ntrees^e^** | **sample_rate^f^** | **reg_alpha^g^** | **reg_lambda^h^** |
| --- | --- | --- | --- | --- | --- | --- | --- | --- | --- |
| Expiratory Reserve Volume (ERV) | XGBoost | 1 | 0.8 | 6 | 20 | 153 | 0.8 | 0.01 | 100 |
| Functional Residual Capacity (FRC) | XGBoost | 1 | 0.8 | 6 | 1 | 470 | 1.0 | 0.01 | 1 |
| RV / TLC | XGBoost | 1 | 0.8 | 6 | 20 | 320 | 0.8 | 0.01 | 100 |
| Residual Volume (RV) | XGBoost | 1 | 0.8 | 6 | 20 | 296 | 0.8 | 0.01 | 100 |
| Total Lung Capacity (TLC) | GBM | 1 | 1.0 | 7 | 10 | 74 | 0.7 |  |  |
| Vital Capacity (VC) | XGBoost | 1 | 1.0 | 6 | 10 | 425 | 1.0 | 0.50 | 1 |
| ^a^Fraction of columns sampled for each tree; ^b^Fraction of columns sampled for each split; ^c^Maximum depth of each tree; ^d^Minimum number of observations in a terminal node; ^e^Number of trees; ^f^Fraction of the training data sampled for each tree; ^g^L1 regularization of model weights (XGBoost); ^h^L2 regularization of model weights (XGBoost); | | | | | | | | | |
