# Supplementary material for: Estimation of Static Lung Volumes and Capacities From Spirometry Using Machine Learning: Algorithm Development and Validation
Source: JMIR AI. 2025 Mar 24;4:e65456. doi: 10.2196/65456 (PMC12223454; doi:10.2196/65456)
Supplement: Multimedia Appendix 3 [file ai-v4-e65456-s003.docx]

|  | **Normal (N=14346)** | **Obstruction (N=7173)** | **Restriction (N=8482)** | **Mixed Defect (N=4512)** | **Total (N=34513)** | **P value** |
| --- | --- | --- | --- | --- | --- | --- |
| **Age (yrs)** | 65.4  (18.0, 95.7) | 65.8  (18.0, 95.7) | 62.7  (18.0, 95.8) | 65.0  (18.0, 94.9) | 64.7  (18.0, 95.8) | <0.001 |
| **Gender** |  |  |  |  |  | <0.001 |
| **Female** | 7078 (49.3%) | 3252 (45.3%) | 4132 (48.7%) | 2108 (46.7%) | 16570 (48.0%) |  |
| **Male** | 7268 (50.7%) | 3921 (54.7%) | 4350 (51.3%) | 2404 (53.3%) | 17943 (52.0%) |  |
| **Race** |  |  |  |  |  | <0.001 |
| **Caucasian** | 13353 (93.1%) | 6892 (96.1%) | 7895 (93.1%) | 4308 (95.5%) | 32448 (94.0%) |  |
| **African American** | 719 (5.0%) | 142 (2.0%) | 400 (4.7%) | 122 (2.7%) | 1383 (4.0%) |  |
| **SE Asian** | 92 (0.6%) | 45 (0.6%) | 48 (0.6%) | 17 (0.4%) | 202 (0.6%) |  |
| **NE Asian** | 7 (0.0%) | 8 (0.1%) | 8 (0.1%) | 4 (0.1%) | 27 (0.1%) |  |
| **Other** | 175 (1.2%) | 86 (1.2%) | 131 (1.5%) | 61 (1.4%) | 453 (1.3%) |  |
| **Height** | 1.7  (1.1, 2.0) | 1.7  (0.2, 2.0) | 1.7  (1.2, 2.0) | 1.7  (1.3, 2.0) | 1.7  (0.2, 2.0) | <0.001 |
| **Weight** | 83.4  (27.0, 202.8) | 78.6  (34.5, 400.0) | 87.5 (12.9, 242.0) | 80.0 (31.0, 233.0) | 82.8 (12.9, 400.0) | <0.001 |
| **FEV1^a^** | 2.6 (0.9, 6.1) | 1.9 (0.5, 5.5) | 1.7 (0.4, 4.6) | 1.1 (0.2, 3.3) | 2.0 (0.2, 6.1) | <0.001 |
| **FVC^b^** | 3.4 (1.1, 7.8) | 3.5 (1.2, 8.3) | 2.2 (0.5, 5.4) | 2.2 (0.6, 5.1) | 2.9 (0.5, 8.3) | <0.001 |
| **FEV1/FVC^c^** | 77.1 (58.7, 100.0) | 58.8 (17.2, 80.5) | 77.2 (58.7, 100.0) | 52.6 (16.2, 76.7) | 71.8 (16.2, 100.0) | <0.001 |
| **PEF^d^** | 7.5 (1.9, 17.5) | 5.6 (0.6, 14.6) | 5.7 (0.9, 15.0) | 3.5 (0.7, 11.7) | 6.2 (0.6, 17.5) | <0.001 |
| **VC (Spiro)^e^** | 3.4 (1.1, 7.8) | 3.5 (1.2, 8.3) | 2.3 (0.5, 5.5) | 2.2 (0.6, 5.2) | 3.0 (0.5, 8.3) | <0.001 |
| **RV^f^** | 2.1 (0.4, 5.8) | 2.9 (0.6, 8.9) | 1.9 (0.1, 7.4) | 3.5 (0.5, 10.4) | 2.3 (0.1, 10.4) | <0.001 |
| **TLC^g^** | 5.5 (2.3, 12.3) | 6.6 (2.8, 13.1) | 4.3 (1.3, 9.7) | 5.9 (1.8, 12.4) | 5.5 (1.3, 13.1) | <0.001 |
| **RV/TLC^h^** | 38.2 (11.3, 73.6) | 46.0 (12.3, 79.1) | 45.5 (3.4, 85.6) | 60.1 (9.2, 89.7) | 43.5 (3.4, 89.7) | <0.001 |
| **FRC^i^** | 3.0 (1.1, 7.5) | 4.0 (1.6, 10.5) | 2.5 (0.4, 8.5) | 4.2 (1.1, 10.8) | 3.2 (0.4, 10.8) | <0.001 |
| **ERV^j^** | 0.9 (0.0, 4.1) | 1.1 (0.0, 3.9) | 0.5 (0.0, 3.5) | 0.7 (0.0, 3.6) | 0.8 (0.0, 4.1) | <0.001 |
| **VC (Pleth)^k^** | 3.4 (1.2, 8.3) | 3.6 (1.2, 8.4) | 2.3 (0.5, 5.8) | 2.3 (0.6, 5.2) | 3.0 (0.5, 8.4) | <0.001 |
| ^a^Forced expiratory volume in the first second; ^b^Forced vital capacity; ^c^Ratio of FEV1 to FVC (as a percentage); ^d^Peak expiratory flow; ^e^Vital capacity measured via spirometry; ^f^Residual volume; ^g^Total lung capacity; ^h^Ratio of RV to TLC (as a percentage); ^i^Functional residual capacity; ^j^Expiratory reserve volume; ^k^Vital capacity measured via body plethysmography | | | | | | |
