# Supplementary material for: Estimation of Static Lung Volumes and Capacities From Spirometry Using Machine Learning: Algorithm Development and Validation
Source: JMIR AI. 2025 Mar 24;4:e65456. doi: 10.2196/65456 (PMC12223454; doi:10.2196/65456)
Supplement: Multimedia Appendix 4 [file ai-v4-e65456-s004.docx]

|  | Train (N=80062) | Test (N=34313) | Total (N=114375) | Standardized Difference |
| --- | --- | --- | --- | --- |
| Age | 63.2 (18.0, 81.0) | 63.1 (18.0, 119.6) | 63.2 (18.0, 119.6) | 0.005 |
| Sex |  |  |  | 0.002 |
| F | 39267 (49.0%) | 16798 (49.0%) | 56065 (49.0%) |  |
| M | 40795 (51.0%) | 17515 (51.0%) | 58310 (51.0%) |  |
| Race |  |  |  | 0.008 |
| Caucasian | 75142 (93.9%) | 32164 (93.7%) | 107306 (93.8%) |  |
| African American | 3239 (4.0%) | 1414 (4.1%) | 4653 (4.1%) |  |
| SE Asian | 491 (0.6%) | 219 (0.6%) | 710 (0.6%) |  |
| NE Asian | 56 (0.1%) | 30 (0.1%) | 86 (0.1%) |  |
| Other | 1134 (1.4%) | 486 (1.4%) | 1620 (1.4%) |  |
| Height | 1.7 (0.2, 2.1) | 1.7 (1.3, 2.2) | 1.7 (0.2, 2.2) | <0.001 |
| Weight | 83.6 (7.8, 400.0) | 83.6 (26.0, 242.0) | 83.6 (7.8, 400.0) | 0.003 |
| ATS Pattern |  |  |  | 0.012 |
| Normal | 31099 (41.1%) | 13190 (40.6%) | 44289 (40.9%) |  |
| Obstruction | 15522 (20.5%) | 6641 (20.4%) | 22163 (20.5%) |  |
| Restriction | 19064 (25.2%) | 8292 (25.5%) | 27356 (25.3%) |  |
| Mixed Defect | 10056 (13.3%) | 4379 (13.5%) | 14435 (13.3%) |  |
| FEV1^a^ | 2.1 (0.2, 6.8) | 2.1 (0.2, 6.2) | 2.1 (0.2, 6.8) | 0.006 |
| FVC^b^ | 3.0 (0.3, 8.8) | 2.9 (0.3, 8.2) | 3.0 (0.3, 8.8) | 0.006 |
| FEV1/FVC^c^ | 72.1 (16.2, 100.0) | 72.1 (18.1, 100.0) | 72.1 (16.2, 100.0) | 0.001 |
| PEF^d^ | 6.3 (0.6, 18.8) | 6.2 (0.6, 18.8) | 6.3 (0.6, 18.8) | 0.002 |
| FET^e^ | 8.8 (-2.6, 89.2) | 8.8 (0.4, 32.3) | 8.8 (-2.6, 89.2) | 0.014 |
| VC (Spiro)^f^ | 3.0 (0.3, 8.8) | 3.0 (0.3, 8.2) | 3.0 (0.3, 8.8) | 0.006 |
| RV^g^ |  |  |  | 0.001 |
| Normal | 59419 (75.6%) | 25470 (75.7%) | 84889 (75.6%) |  |
| Abnormal | 19150 (24.4%) | 8195 (24.3%) | 27345 (24.4%) |  |
| TLC^h^ |  |  |  | 0.016 |
| Abnormal | 24850 (31.6%) | 10901 (32.4%) | 35751 (31.9%) |  |
| Normal | 53719 (68.4%) | 22764 (67.6%) | 76483 (68.1%) |  |
| RV/TLC^i^ |  |  |  | 0.011 |
| Normal | 44544 (56.7%) | 18904 (56.2%) | 63448 (56.5%) |  |
| Abnormal | 34024 (43.3%) | 14761 (43.8%) | 48785 (43.5%) |  |
| FRC^j^ |  |  |  | <0.001 |
| Abnormal | 20254 (25.8%) | 8676 (25.8%) | 28930 (25.8%) |  |
| Normal | 58340 (74.2%) | 24996 (74.2%) | 83336 (74.2%) |  |
| ERV^k^ |  |  |  | 0.001 |
| Normal | 66272 (84.4%) | 28382 (84.3%) | 94654 (84.3%) |  |
| Abnormal | 12295 (15.6%) | 5282 (15.7%) | 17577 (15.7%) |  |
| VC (Pleth)^l^ |  |  |  | 0.013 |
| Normal | 35216 (44.6%) | 14866 (43.9%) | 50082 (44.4%) |  |
| Abnormal | 43748 (55.4%) | 18965 (56.1%) | 62713 (55.6%) |  |
| ^a^Forced expiratory volume in the first second; ^b^Forced vital capacity; ^c^Ratio of FEV1 to FVC (as a percentage); ^d^Peak expiratory flow; ^e^Forced expiratory time; ^f^Vital capcity measured via spirometry; ^g^Residual volume; ^h^Total lung capacity; ^i^Ratio of RV to TLC (as a percentage); ^j^Functional residual capacity; ^k^Expiratory reserve volume; ^l^Vital capacity measured via body plethysmography | | | | |
