# Supplementary material for: Estimation of Static Lung Volumes and Capacities From Spirometry Using Machine Learning: Algorithm Development and Validation
Source: JMIR AI. 2025 Mar 24;4:e65456. doi: 10.2196/65456 (PMC12223454; doi:10.2196/65456)
Supplement: Multimedia Appendix 5 [file ai-v4-e65456-s005.docx]

|  | Normal (N=44289) | Obstruction (N=22163) | Restriction (N=27356) | Mixed Defect (N=14435) | Total (N=108243) | P value |
| --- | --- | --- | --- | --- | --- | --- |
| Age | 63.6 (18.0, 119.6) | 64.1 (18.0, 81.0) | 61.4 (18.0, 81.0) | 64.1 (18.0, 81.0) | 63.2 (18.0, 119.6) | <0.001 |
| Sex |  |  |  |  |  | <0.001 |
| F | 22307 (50.4%) | 10181 (45.9%) | 13421 (49.1%) | 6800 (47.1%) | 52709 (48.7%) |  |
| M | 21982 (49.6%) | 11982 (54.1%) | 13935 (50.9%) | 7635 (52.9%) | 55534 (51.3%) |  |
| Race |  |  |  |  |  | <0.001 |
| Caucasian | 40932 (92.4%) | 21256 (95.9%) | 25403 (92.9%) | 13809 (95.7%) | 101400 (93.7%) |  |
| African American | 2422 (5.5%) | 462 (2.1%) | 1266 (4.6%) | 368 (2.5%) | 4518 (4.2%) |  |
| SE Asian | 310 (0.7%) | 162 (0.7%) | 155 (0.6%) | 49 (0.3%) | 676 (0.6%) |  |
| NE Asian | 34 (0.1%) | 15 (0.1%) | 27 (0.1%) | 9 (0.1%) | 85 (0.1%) |  |
| Other | 591 (1.3%) | 268 (1.2%) | 505 (1.8%) | 200 (1.4%) | 1564 (1.4%) |  |
| Height | 1.7 (1.1, 2.1) | 1.7 (0.2, 2.1) | 1.7 (1.2, 2.2) | 1.7 (1.3, 2.0) | 1.7 (0.2, 2.2) | <0.001 |
| Weight | 84.1 (27.0, 234.4) | 79.0 (31.2, 400.0) | 88.2 (7.8, 253.4) | 80.4 (26.0, 233.0) | 83.4 (7.8, 400.0) | <0.001 |
| FEV1^a^ | 2.6 (1.1, 6.8) | 2.0 (0.4, 5.7) | 1.7 (0.3, 5.1) | 1.1 (0.2, 3.7) | 2.1 (0.2, 6.8) | <0.001 |
| FVC^b^ | 3.4 (1.3, 8.8) | 3.5 (1.2, 8.7) | 2.3 (0.3, 6.1) | 2.2 (0.3, 5.5) | 3.0 (0.3, 8.8) | <0.001 |
| FEV1/FVC^c^ | 77.5 (60.0, 100.0) | 59.4 (16.2, 80.5) | 77.4 (59.8, 100.0) | 53.2 (16.2, 78.3) | 72.4 (16.2, 100.0) | <0.001 |
| PEF^d^ | 7.6 (1.9, 18.8) | 5.7 (0.6, 18.8) | 5.8 (0.7, 15.0) | 3.6 (0.6, 12.9) | 6.3 (0.6, 18.8) | <0.001 |
| FET^e^ | 8.2 (0.5, 31.8) | 11.2 (-0.6, 47.3) | 7.7 (-0.0, 89.2) | 9.9 (-2.6, 31.8) | 8.7 (-2.6, 89.2) | <0.001 |
| VC (Spiro)^f^ | 3.5 (1.3, 8.8) | 3.6 (1.2, 8.7) | 2.3 (0.3, 6.4) | 2.3 (0.3, 5.5) | 3.0 (0.3, 8.8) | <0.001 |
| RV^g^ |  |  |  |  |  | <0.001 |
| Normal | 39801 (92.3%) | 12026 (54.5%) | 23212 (86.8%) | 4755 (33.3%) | 79794 (75.1%) |  |
| Abnormal | 3315 (7.7%) | 10054 (45.5%) | 3531 (13.2%) | 9526 (66.7%) | 26426 (24.9%) |  |
| TLC^h^ |  |  |  |  |  | <0.001 |
| Abnormal | 7442 (17.3%) | 4579 (20.7%) | 18223 (68.1%) | 4825 (33.8%) | 35069 (33.0%) |  |
| Normal | 35674 (82.7%) | 17501 (79.3%) | 8520 (31.9%) | 9456 (66.2%) | 71151 (67.0%) |  |
| RV/TLC^i^ |  |  |  |  |  | <0.001 |
| Normal | 37648 (87.3%) | 10409 (47.1%) | 11356 (42.5%) | 813 (5.7%) | 60226 (56.7%) |  |
| Abnormal | 5467 (12.7%) | 11671 (52.9%) | 15387 (57.5%) | 13468 (94.3%) | 45993 (43.3%) |  |
| FRC^j^ |  |  |  |  |  | <0.001 |
| Abnormal | 5466 (12.7%) | 7851 (35.6%) | 8157 (30.5%) | 6953 (48.7%) | 28427 (26.8%) |  |
| Normal | 37664 (87.3%) | 14232 (64.4%) | 18598 (69.5%) | 7331 (51.3%) | 77825 (73.2%) |  |
| ERV^k^ |  |  |  |  |  | <0.001 |
| Normal | 38920 (90.3%) | 20439 (92.6%) | 18490 (69.1%) | 11717 (82.0%) | 89566 (84.3%) |  |
| Abnormal | 4194 (9.7%) | 1641 (7.4%) | 8253 (30.9%) | 2564 (18.0%) | 16652 (15.7%) |  |
| VC (Pleth)^l^ |  |  |  |  |  | <0.001 |
| Normal | 30841 (70.8%) | 16872 (76.4%) | 314 (1.2%) | 322 (2.2%) | 48349 (45.3%) |  |
| Abnormal | 12745 (29.2%) | 5218 (23.6%) | 26472 (98.8%) | 13992 (97.8%) | 58427 (54.7%) |  |
| ^a^Forced expiratory volume in the first second; ^b^Forced vital capacity; ^c^Ratio of FEV1 to FVC (as a percentage); ^d^Peak expiratory flow; ^e^Forced expiratory time; ^f^Vital capcity measured via spirometry; ^g^Residual volume; ^h^Total lung capacity; ^i^Ratio of RV to TLC (as a percentage); ^j^Functional residual capacity; ^k^Expiratory reserve volume; ^l^Vital capacity measured via body plethysmography | | | | | | |
