# Supplementary material for: Estimation of Static Lung Volumes and Capacities From Spirometry Using Machine Learning: Algorithm Development and Validation
Source: JMIR AI. 2025 Mar 24;4:e65456. doi: 10.2196/65456 (PMC12223454; doi:10.2196/65456)
Supplement: Multimedia Appendix 6 [file ai-v4-e65456-s006.docx]

| **Volume** | **ATS Pattern** | **Dataset** |  | **RMSE^a^** |  | **MAE^b^** |  | **MSD^c^** |  | **MPE^d^** |  | **MAPE^e^** |  | **Rsq^f^** |
| --- | --- | --- | --- | --- | --- | --- | --- | --- | --- | --- | --- | --- | --- | --- |
| Expiratory Reserve Volume (ERV) | Overall | Train |  | 0.31 |  | 0.24 |  | 0 |  | -40.12 |  | 60.28 |  | 0.64 |
|  |  | Test |  | 0.33 |  | 0.25 |  | 0 |  | -39.1 |  | 59.95 |  | 0.61 |
|  | Normal | Train |  | 0.33 |  | 0.25 |  | 0 |  | -35.84 |  | 55.01 |  | 0.61 |
|  |  | Test |  | 0.34 |  | 0.26 |  | 0 |  | -27.62 |  | 47.01 |  | 0.59 |
|  | Obstruction | Train |  | 0.36 |  | 0.27 |  | 0 |  | -21.16 |  | 39.4 |  | 0.62 |
|  |  | Test |  | 0.38 |  | 0.29 |  | 0.01 |  | -22.63 |  | 42.13 |  | 0.58 |
|  | Restriction | Train |  | 0.26 |  | 0.2 |  | 0 |  | -64.12 |  | 86.96 |  | 0.52 |
|  |  | Test |  | 0.27 |  | 0.21 |  | 0.01 |  | -70.57 |  | 94.61 |  | 0.46 |
|  | Mixed Defect | Train |  | 0.29 |  | 0.22 |  | 0 |  | -40.08 |  | 60.97 |  | 0.5 |
|  |  | Test |  | 0.31 |  | 0.23 |  | 0 |  | -41.9 |  | 63.86 |  | 0.43 |
| Functional Residual Capacity (FRC) | Overall | Train |  | 0.56 |  | 0.42 |  | 0 |  | -2.83 |  | 12.93 |  | 0.78 |
|  |  | Test |  | 0.59 |  | 0.44 |  | 0 |  | -2.91 |  | 13.51 |  | 0.75 |
|  | Normal | Train |  | 0.46 |  | 0.35 |  | 0 |  | -2.22 |  | 11.68 |  | 0.7 |
|  |  | Test |  | 0.48 |  | 0.37 |  | 0.01 |  | -2.2 |  | 12.15 |  | 0.68 |
|  | Obstruction | Train |  | 0.62 |  | 0.48 |  | 0 |  | -2.42 |  | 11.83 |  | 0.7 |
|  |  | Test |  | 0.66 |  | 0.51 |  | 0.01 |  | -2.2 |  | 12.29 |  | 0.65 |
|  | Restriction | Train |  | 0.5 |  | 0.38 |  | 0 |  | -3.7 |  | 14.8 |  | 0.6 |
|  |  | Test |  | 0.52 |  | 0.39 |  | 0 |  | -4.09 |  | 15.71 |  | 0.56 |
|  | Mixed Defect | Train |  | 0.8 |  | 0.61 |  | 0 |  | -3.88 |  | 15.09 |  | 0.73 |
|  |  | Test |  | 0.85 |  | 0.65 |  | -0.01 |  | -4.27 |  | 15.96 |  | 0.66 |
| Residual Volume (RV) | Overall | Train |  | 0.54 |  | 0.4 |  | 0 |  | -4.86 |  | 17.29 |  | 0.73 |
|  |  | Test |  | 0.56 |  | 0.41 |  | 0 |  | -4.92 |  | 17.8 |  | 0.71 |
|  | Normal | Train |  | 0.43 |  | 0.32 |  | 0 |  | -4.05 |  | 16.07 |  | 0.59 |
|  |  | Test |  | 0.44 |  | 0.33 |  | 0.01 |  | -3.69 |  | 16.31 |  | 0.58 |
|  | Obstruction | Train |  | 0.6 |  | 0.46 |  | 0 |  | -4.19 |  | 15.96 |  | 0.62 |
|  |  | Test |  | 0.63 |  | 0.48 |  | 0 |  | -4.16 |  | 16.33 |  | 0.59 |
|  | Restriction | Train |  | 0.5 |  | 0.37 |  | -0.01 |  | -6.79 |  | 19.84 |  | 0.52 |
|  |  | Test |  | 0.51 |  | 0.38 |  | -0.02 |  | -7.73 |  | 21.12 |  | 0.49 |
|  | Mixed Defect | Train |  | 0.81 |  | 0.61 |  | 0.01 |  | -5.18 |  | 18.7 |  | 0.66 |
|  |  | Test |  | 0.83 |  | 0.63 |  | 0 |  | -5.45 |  | 19.22 |  | 0.61 |
| Total Lung Capacity (TLC) | Overall | Train |  | 0.55 |  | 0.41 |  | 0 |  | -1.07 |  | 7.57 |  | 0.87 |
|  |  | Test |  | 0.58 |  | 0.43 |  | 0 |  | -1.1 |  | 7.92 |  | 0.85 |
|  | Normal | Train |  | 0.44 |  | 0.33 |  | 0 |  | -0.63 |  | 6.05 |  | 0.88 |
|  |  | Test |  | 0.45 |  | 0.35 |  | 0.01 |  | -0.48 |  | 6.24 |  | 0.87 |
|  | Obstruction | Train |  | 0.6 |  | 0.46 |  | 0 |  | -0.81 |  | 6.96 |  | 0.83 |
|  |  | Test |  | 0.65 |  | 0.49 |  | 0.01 |  | -0.77 |  | 7.37 |  | 0.8 |
|  | Restriction | Train |  | 0.52 |  | 0.39 |  | 0 |  | -1.56 |  | 9.03 |  | 0.78 |
|  |  | Test |  | 0.54 |  | 0.4 |  | -0.01 |  | -1.85 |  | 9.55 |  | 0.77 |
|  | Mixed Defect | Train |  | 0.81 |  | 0.62 |  | -0.01 |  | -2.11 |  | 10.66 |  | 0.75 |
|  |  | Test |  | 0.86 |  | 0.66 |  | -0.02 |  | -2.42 |  | 11.31 |  | 0.7 |
| RV / TLC (%) | Overall | Train |  | 5.07 |  | 3.93 |  | 0 |  | -1.61 |  | 9.55 |  | 0.82 |
|  |  | Test |  | 5.2 |  | 4.03 |  | 0.03 |  | -1.58 |  | 9.83 |  | 0.81 |
|  | Normal | Train |  | 4.66 |  | 3.61 |  | 0 |  | -1.75 |  | 10.04 |  | 0.73 |
|  |  | Test |  | 4.77 |  | 3.7 |  | 0.07 |  | -1.57 |  | 10.27 |  | 0.71 |
|  | Obstruction | Train |  | 4.82 |  | 3.79 |  | -0.04 |  | -1.39 |  | 8.87 |  | 0.79 |
|  |  | Test |  | 4.89 |  | 3.85 |  | 0.01 |  | -1.35 |  | 9.03 |  | 0.79 |
|  | Restriction | Train |  | 5.66 |  | 4.38 |  | -0.06 |  | -2.01 |  | 10.26 |  | 0.71 |
|  |  | Test |  | 5.95 |  | 4.57 |  | -0.12 |  | -2.35 |  | 10.91 |  | 0.67 |
|  | Mixed Defect | Train |  | 5.5 |  | 4.3 |  | 0.08 |  | -0.95 |  | 7.86 |  | 0.75 |
|  |  | Test |  | 5.6 |  | 4.35 |  | 0.13 |  | -0.9 |  | 7.97 |  | 0.74 |
| Vital Capacity (VC) | Overall | Train |  | 0.15 |  | 0.11 |  | 0 |  | -0.27 |  | 3.73 |  | 0.98 |
|  |  | Test |  | 0.15 |  | 0.11 |  | 0 |  | -0.33 |  | 3.91 |  | 0.98 |
|  | Normal | Train |  | 0.14 |  | 0.11 |  | 0 |  | -0.19 |  | 3.12 |  | 0.98 |
|  |  | Test |  | 0.15 |  | 0.11 |  | 0 |  | -0.23 |  | 3.28 |  | 0.98 |
|  | Obstruction | Train |  | 0.16 |  | 0.12 |  | 0 |  | -0.27 |  | 3.46 |  | 0.98 |
|  |  | Test |  | 0.17 |  | 0.13 |  | 0 |  | -0.35 |  | 3.62 |  | 0.98 |
|  | Restriction | Train |  | 0.14 |  | 0.1 |  | 0 |  | -0.35 |  | 4.2 |  | 0.97 |
|  |  | Test |  | 0.14 |  | 0.1 |  | 0 |  | -0.35 |  | 4.35 |  | 0.97 |
|  | Mixed Defect | Train |  | 0.17 |  | 0.13 |  | 0 |  | -0.36 |  | 5.33 |  | 0.95 |
|  |  | Test |  | 0.18 |  | 0.13 |  | 0 |  | -0.63 |  | 5.62 |  | 0.94 |
| ^a^Root mean squared error; ^b^Mean absolute error; ^c^Mean signed deviation; ^d^Mean percent error; ^e^Mean absolute percent error; ^f^R-Squared | | | | | | | | | | | | | | |
