# Supplementary material for: Estimation of Static Lung Volumes and Capacities From Spirometry Using Machine Learning: Algorithm Development and Validation
Source: JMIR AI. 2025 Mar 24;4:e65456. doi: 10.2196/65456 (PMC12223454; doi:10.2196/65456)
Supplement: Multimedia Appendix 7 [file ai-v4-e65456-s007.docx]

| **Volume** | **ATS Pattern** | **Dataset** |  | **AUC^a^** |  | **ACC^b^** |  | **SENS/REC^c^** |  | **SPEC^d^** |  | **PPV/PRE^e^** |  | **NPV^f^** |  | **LRT+^g^** |  | **LRT-^h^** |  | **OR^i^** |  | **F1^j^** |
| --- | --- | --- | --- | --- | --- | --- | --- | --- | --- | --- | --- | --- | --- | --- | --- | --- | --- | --- | --- | --- | --- | --- |
| Expiratory Reserve Volume (ERV) | Overall | Train |  | 0.86 |  | 0.76 |  | 0.78 |  | 0.76 |  | 0.38 |  | 0.95 |  | 3.28 |  | 0.29 |  | 11.43 |  | 0.51 |
|  |  | Test |  | 0.82 |  | 0.75 |  | 0.73 |  | 0.75 |  | 0.35 |  | 0.94 |  | 2.93 |  | 0.35 |  | 8.26 |  | 0.48 |
|  | Normal | Train |  | 0.83 |  | 0.83 |  | 0.6 |  | 0.85 |  | 0.31 |  | 0.95 |  | 4.06 |  | 0.47 |  | 8.62 |  | 0.4 |
|  |  | Test |  | 0.78 |  | 0.81 |  | 0.53 |  | 0.84 |  | 0.26 |  | 0.94 |  | 3.3 |  | 0.56 |  | 5.89 |  | 0.35 |
|  | Obstruction | Train |  | 0.81 |  | 0.89 |  | 0.42 |  | 0.93 |  | 0.33 |  | 0.95 |  | 6.09 |  | 0.63 |  | 9.7 |  | 0.37 |
|  |  | Test |  | 0.72 |  | 0.88 |  | 0.3 |  | 0.93 |  | 0.25 |  | 0.94 |  | 4.14 |  | 0.75 |  | 5.51 |  | 0.27 |
|  | Restriction | Train |  | 0.83 |  | 0.57 |  | 0.94 |  | 0.41 |  | 0.41 |  | 0.94 |  | 1.6 |  | 0.15 |  | 10.4 |  | 0.57 |
|  |  | Test |  | 0.77 |  | 0.56 |  | 0.91 |  | 0.4 |  | 0.41 |  | 0.91 |  | 1.53 |  | 0.22 |  | 6.94 |  | 0.57 |
|  | Mixed Defect | Train |  | 0.86 |  | 0.73 |  | 0.83 |  | 0.71 |  | 0.39 |  | 0.95 |  | 2.84 |  | 0.24 |  | 11.7 |  | 0.53 |
|  |  | Test |  | 0.8 |  | 0.7 |  | 0.75 |  | 0.68 |  | 0.34 |  | 0.93 |  | 2.39 |  | 0.36 |  | 6.6 |  | 0.46 |
| Functional Residual Capacity (FRC) | Overall | Train |  | 0.88 |  | 0.79 |  | 0.8 |  | 0.79 |  | 0.58 |  | 0.92 |  | 3.8 |  | 0.25 |  | 14.98 |  | 0.67 |
|  |  | Test |  | 0.84 |  | 0.77 |  | 0.76 |  | 0.77 |  | 0.55 |  | 0.9 |  | 3.32 |  | 0.31 |  | 10.65 |  | 0.64 |
|  | Normal | Train |  | 0.85 |  | 0.87 |  | 0.52 |  | 0.92 |  | 0.49 |  | 0.93 |  | 6.6 |  | 0.52 |  | 12.7 |  | 0.5 |
|  |  | Test |  | 0.79 |  | 0.86 |  | 0.47 |  | 0.91 |  | 0.44 |  | 0.92 |  | 5.36 |  | 0.58 |  | 9.22 |  | 0.45 |
|  | Obstruction | Train |  | 0.86 |  | 0.73 |  | 0.87 |  | 0.65 |  | 0.58 |  | 0.9 |  | 2.46 |  | 0.21 |  | 11.87 |  | 0.69 |
|  |  | Test |  | 0.83 |  | 0.71 |  | 0.83 |  | 0.64 |  | 0.55 |  | 0.87 |  | 2.29 |  | 0.26 |  | 8.68 |  | 0.66 |
|  | Restriction | Train |  | 0.85 |  | 0.75 |  | 0.81 |  | 0.72 |  | 0.56 |  | 0.9 |  | 2.87 |  | 0.27 |  | 10.8 |  | 0.66 |
|  |  | Test |  | 0.79 |  | 0.71 |  | 0.75 |  | 0.69 |  | 0.51 |  | 0.86 |  | 2.4 |  | 0.36 |  | 6.65 |  | 0.61 |
|  | Mixed Defect | Train |  | 0.89 |  | 0.74 |  | 0.93 |  | 0.56 |  | 0.67 |  | 0.9 |  | 2.13 |  | 0.12 |  | 18.26 |  | 0.78 |
|  |  | Test |  | 0.86 |  | 0.72 |  | 0.92 |  | 0.53 |  | 0.65 |  | 0.87 |  | 1.95 |  | 0.16 |  | 12.26 |  | 0.76 |
| Residual Volume (RV) | Overall | Train |  | 0.9 |  | 0.82 |  | 0.82 |  | 0.83 |  | 0.61 |  | 0.93 |  | 4.69 |  | 0.22 |  | 20.97 |  | 0.7 |
|  |  | Test |  | 0.87 |  | 0.8 |  | 0.77 |  | 0.81 |  | 0.57 |  | 0.91 |  | 4.01 |  | 0.29 |  | 13.96 |  | 0.66 |
|  | Normal | Train |  | 0.82 |  | 0.92 |  | 0.28 |  | 0.97 |  | 0.46 |  | 0.94 |  | 10.62 |  | 0.74 |  | 14.37 |  | 0.35 |
|  |  | Test |  | 0.78 |  | 0.91 |  | 0.2 |  | 0.97 |  | 0.36 |  | 0.93 |  | 6.33 |  | 0.82 |  | 7.68 |  | 0.26 |
|  | Obstruction | Train |  | 0.84 |  | 0.65 |  | 0.94 |  | 0.41 |  | 0.57 |  | 0.9 |  | 1.59 |  | 0.14 |  | 11.48 |  | 0.71 |
|  |  | Test |  | 0.77 |  | 0.62 |  | 0.9 |  | 0.39 |  | 0.55 |  | 0.82 |  | 1.47 |  | 0.26 |  | 5.72 |  | 0.68 |
|  | Restriction | Train |  | 0.81 |  | 0.85 |  | 0.47 |  | 0.91 |  | 0.45 |  | 0.92 |  | 5.34 |  | 0.58 |  | 9.24 |  | 0.46 |
|  |  | Test |  | 0.74 |  | 0.82 |  | 0.37 |  | 0.89 |  | 0.33 |  | 0.9 |  | 3.32 |  | 0.71 |  | 4.68 |  | 0.35 |
|  | Mixed Defect | Train |  | 0.88 |  | 0.74 |  | 0.99 |  | 0.22 |  | 0.72 |  | 0.9 |  | 1.27 |  | 0.05 |  | 24.15 |  | 0.83 |
|  |  | Test |  | 0.85 |  | 0.71 |  | 0.98 |  | 0.18 |  | 0.7 |  | 0.85 |  | 1.2 |  | 0.09 |  | 13.29 |  | 0.82 |
| Total Lung Capacity (TLC) | Overall | Train |  | 0.93 |  | 0.85 |  | 0.85 |  | 0.84 |  | 0.73 |  | 0.92 |  | 5.42 |  | 0.18 |  | 30.91 |  | 0.78 |
|  |  | Test |  | 0.9 |  | 0.81 |  | 0.8 |  | 0.82 |  | 0.69 |  | 0.89 |  | 4.48 |  | 0.24 |  | 18.69 |  | 0.74 |
|  | Normal | Train |  | 0.93 |  | 0.9 |  | 0.73 |  | 0.93 |  | 0.68 |  | 0.94 |  | 10.55 |  | 0.29 |  | 36.24 |  | 0.71 |
|  |  | Test |  | 0.9 |  | 0.88 |  | 0.67 |  | 0.92 |  | 0.65 |  | 0.93 |  | 8.63 |  | 0.36 |  | 24.17 |  | 0.66 |
|  | Obstruction | Train |  | 0.88 |  | 0.84 |  | 0.67 |  | 0.89 |  | 0.61 |  | 0.91 |  | 5.98 |  | 0.38 |  | 15.93 |  | 0.64 |
|  |  | Test |  | 0.81 |  | 0.81 |  | 0.56 |  | 0.87 |  | 0.53 |  | 0.88 |  | 4.32 |  | 0.51 |  | 8.47 |  | 0.54 |
|  | Restriction | Train |  | 0.91 |  | 0.81 |  | 0.96 |  | 0.47 |  | 0.8 |  | 0.86 |  | 1.82 |  | 0.08 |  | 23.32 |  | 0.87 |
|  |  | Test |  | 0.87 |  | 0.78 |  | 0.94 |  | 0.44 |  | 0.78 |  | 0.79 |  | 1.69 |  | 0.13 |  | 13.28 |  | 0.85 |
|  | Mixed Defect | Train |  | 0.86 |  | 0.77 |  | 0.8 |  | 0.75 |  | 0.62 |  | 0.88 |  | 3.24 |  | 0.27 |  | 12.02 |  | 0.69 |
|  |  | Test |  | 0.77 |  | 0.71 |  | 0.72 |  | 0.7 |  | 0.57 |  | 0.82 |  | 2.4 |  | 0.4 |  | 5.99 |  | 0.63 |
| RV / TLC (%) | Overall | Train |  | 0.91 |  | 0.83 |  | 0.82 |  | 0.84 |  | 0.8 |  | 0.86 |  | 5.16 |  | 0.21 |  | 24.5 |  | 0.81 |
|  |  | Test |  | 0.91 |  | 0.82 |  | 0.81 |  | 0.83 |  | 0.79 |  | 0.85 |  | 4.8 |  | 0.23 |  | 21.13 |  | 0.8 |
|  | Normal | Train |  | 0.83 |  | 0.88 |  | 0.21 |  | 0.98 |  | 0.61 |  | 0.9 |  | 10.98 |  | 0.81 |  | 13.58 |  | 0.31 |
|  |  | Test |  | 0.81 |  | 0.88 |  | 0.18 |  | 0.98 |  | 0.54 |  | 0.89 |  | 8.07 |  | 0.84 |  | 9.65 |  | 0.27 |
|  | Obstruction | Train |  | 0.87 |  | 0.78 |  | 0.86 |  | 0.69 |  | 0.76 |  | 0.82 |  | 2.76 |  | 0.2 |  | 14.02 |  | 0.81 |
|  |  | Test |  | 0.85 |  | 0.76 |  | 0.84 |  | 0.67 |  | 0.74 |  | 0.79 |  | 2.53 |  | 0.24 |  | 10.57 |  | 0.79 |
|  | Restriction | Train |  | 0.82 |  | 0.73 |  | 0.86 |  | 0.57 |  | 0.73 |  | 0.75 |  | 2 |  | 0.25 |  | 7.98 |  | 0.79 |
|  |  | Test |  | 0.8 |  | 0.72 |  | 0.84 |  | 0.55 |  | 0.73 |  | 0.72 |  | 1.9 |  | 0.28 |  | 6.76 |  | 0.78 |
|  | Mixed Defect | Train |  | 0.86 |  | 0.95 |  | 1 |  | 0.05 |  | 0.95 |  | 0.81 |  | 1.05 |  | 0.01 |  | 75.26 |  | 0.97 |
|  |  | Test |  | 0.86 |  | 0.94 |  | 1 |  | 0.03 |  | 0.94 |  | 0.78 |  | 1.03 |  | 0.02 |  | 59.51 |  | 0.97 |
| Vital Capacity (VC) | Overall | Train |  | 0.99 |  | 0.95 |  | 0.95 |  | 0.95 |  | 0.96 |  | 0.94 |  | 20.15 |  | 0.05 |  | 393.95 |  | 0.96 |
|  |  | Test |  | 0.98 |  | 0.93 |  | 0.93 |  | 0.94 |  | 0.95 |  | 0.92 |  | 14.48 |  | 0.07 |  | 203.37 |  | 0.94 |
|  | Normal | Train |  | 0.98 |  | 0.93 |  | 0.86 |  | 0.96 |  | 0.91 |  | 0.94 |  | 23.63 |  | 0.14 |  | 163.85 |  | 0.88 |
|  |  | Test |  | 0.97 |  | 0.91 |  | 0.82 |  | 0.95 |  | 0.88 |  | 0.93 |  | 16.95 |  | 0.19 |  | 89 |  | 0.85 |
|  | Obstruction | Train |  | 0.97 |  | 0.92 |  | 0.8 |  | 0.96 |  | 0.86 |  | 0.94 |  | 19.23 |  | 0.21 |  | 90.74 |  | 0.83 |
|  |  | Test |  | 0.94 |  | 0.88 |  | 0.7 |  | 0.94 |  | 0.77 |  | 0.91 |  | 10.86 |  | 0.32 |  | 34.21 |  | 0.74 |
|  | Restriction | Train |  | 0.96 |  | 0.99 |  | 1 |  | 0.31 |  | 0.99 |  | 0.93 |  | 1.46 |  | 0 |  | 1694.76 |  | 1 |
|  |  | Test |  | 0.93 |  | 0.99 |  | 1 |  | 0.2 |  | 0.99 |  | 0.69 |  | 1.25 |  | 0.01 |  | 225.46 |  | 0.99 |
|  | Mixed Defect | Train |  | 0.95 |  | 0.98 |  | 1 |  | 0.24 |  | 0.98 |  | 0.93 |  | 1.32 |  | 0 |  | 778.88 |  | 0.99 |
|  |  | Test |  | 0.84 |  | 0.98 |  | 1 |  | 0.13 |  | 0.98 |  | 0.71 |  | 1.15 |  | 0.01 |  | 129.02 |  | 0.99 |
| ^a^Area under the ROC curve; ^b^Accuracy; ^c^Sensitivity or Recall; ^d^Specificity; ^e^Positive Predictive Value or Precision; ^f^Negative Predictive Value; ^g^Likelihood Ratio Test +; ^h^Likelihood Ratio Test -; ^i^Odds Ratio; ^j^F1 Score; | | | | | | | | | | | | | | | | | | | | | | |
